# Chromosome-Level Genome Assembly of Chinese Sucker (*Myxocyprinus asiaticus*) Reveals Strongly Conserved Synteny Following a Catostomid-Specific Whole-Genome Duplication

**DOI:** 10.1093/gbe/evab190

**Published:** 2021-08-12

**Authors:** Trevor J Krabbenhoft, Daniel J MacGuigan, Nathan J C Backenstose, Hannah Waterman, Tianying Lan, Jessie A Pelosi, Milton Tan, Simen R Sandve

**Affiliations:** 1 Department of Biological Sciences and the RENEW Institute, University at Buffalo, USA; 2 Department of Biological Sciences, University at Buffalo, USA; 3 Department of Biology, University of Florida, USA; 4 Illinois Natural History Survey, University of Illinois at Urbana-Champaign, USA; 5 Department of Animal and Aquacultural Sciences, Faculty of Biosciences, Norwegian University of Life Sciences, Ås, Norway

**Keywords:** polyploidy, genome stability, genome architecture, allopolyploidy, fractionation, fish

## Abstract

Fishes of the family Catostomidae (“suckers”; Teleostei: Cypriniformes) are hypothesized to have undergone an allopolyploidy event approximately 60 Ma. However, genomic evidence has previously been unavailable to assess this hypothesis. We sequenced and assembled the first chromosome-level catostomid genome, Chinese sucker (*Myxocyprinus asiaticus*), and present clear evidence of a catostomid-specific whole-genome duplication (WGD) event (“Cat-4R”). Our results reveal remarkably strong, conserved synteny since this duplication event, as well as between *Myxocyprinus* and an unduplicated outgroup, zebrafish (*Danio rerio*). Gene content and repetitive elements are also approximately evenly distributed across homeologous chromosomes, suggesting that both subgenomes retain some function, with no obvious bias in gene fractionation or subgenome dominance. The Cat-4R duplication provides another independent example of genome evolution following WGD in animals, in this case at the extreme end of conserved genome architecture over at least 25.2 Myr since the duplication. The *M. asiaticus* genome is a useful resource for researchers interested in understanding genome evolution following WGD in animals.

## Introduction

The fish family Catostomidae (“suckers”) is comprised of approximately 80 cypriniform species, of which only one is extant in Asia, with all others being North American endemics. The Asian species, Chinese sucker (*Myxocyprinus asiaticus*) is recognized as the sole member of the catostomid subfamily Myxocyprininae (Harris and Mayden 2001; Tan and Armbruster 2018), and is phylogenetically sister to all other extant sucker species, having diverged an estimated ∼63 Ma (Bagley et al. 2018). Although *M. asiaticus* is the only surviving catostomid species in Asia (aside from secondary colonization by *Catostomus catostomus*), numerous fossil species have been described across both Asia and North America (Miller and Smith, 1981; Grande et al. 1982; Liu and Chang, 2009).

In addition to the teleost-specific whole-genome duplication (WGD) that occurred around 320–350 Ma (Christoffels et al. 2004; Vandepoele et al. 2004), catostomid fishes underwent an additional ancestral WGD event (“Cat-4R”). Initial evidence for this WGD stemmed from karyotypic and allozyme data (Uyeno and Smith 1972; Ferris and Whitt 1977, 1980). The lack of tetrasomic inheritance in allozyme data and chromosome pairing behavior during meiosis was interpreted to imply that Cat-4R was an allopolyploidy event (reviewed in Ferris [1984]). Subsequent literature has generally assumed this to be the case, but without direct genome-wide sequence data to test that hypothesis.

We sequenced and assembled the first chromosome-level catostomid fish genome, Chinese sucker, *M.asiaticus*, to provide a resource with which to evaluate these hypotheses. This assembly complements a growing list of noncatostomid, cypriniform genomes available, including Tibetan loach (*Triplophysa tibetana*; Yang et al. 2019), zebrafish (*Danio rerio*; Howe et al. 2013), common carp (*Cyprinus carpio*; Xu et al. 2014), and other cyprinids. Our primary aims were to 1) characterize genome structure in this species, 2) identify homeologous chromosome pairs and compare duplicated gene and repeat content among homeologs, and 3) evaluate the degree of neutral genetic differentiation (synonymous substitution rates, *K_s_*) between subgenomes and between Chinese sucker and within and between related species.

## Results and Discussion

### DNA and Transcriptome Sequencing

A total of 9,069,422 Oxford Nanopore reads were generated, containing 148.3 Gb of sequence data (approximately 62× genome coverage). Nanopore sequences had a read N50 of ∼37.9 kb, with the longest read >388 kb (supplementary figs. S1 and S2, Supplementary Material online). An additional 202.3 GB (∼80× coverage) of Illumina PE150 Novaseq6000 shotgun genomic reads were generated and used in assembly polishing (supplementary table S1, Supplementary Material online). A total of 281.9 million transcriptome reads were produced across the 12 tissues (84.6 Gb), of which 276.9 million passed QC filtering (83.1 Gb) and were used in de novo transcriptome assembly for gene model evidence (supplementary table S2, Supplementary Material online). These data contained 6.2–7.9 Gb sequence per tissue after filtering.

### Genome Assembly

The initial assembly resulted in a contig N50 of 4.19 MB across 1,910 contigs (table 1). Hi-C scaffolding produced 50 scaffolds ranging from 27.7 to 72.2 Mb in length (supplementary table S3, Supplementary Material online), with scaffold N50 = 49.2 Mb and a final assembly 2.58 Gb in length (within the range of expectation based on flow cytometry; table 1). The final assembly was high quality as evidenced by 98.3% complete BUSCO (table 1 and supplementary fig. S3, Supplementary Material online), and lacked microbial or other contamination based on Kracken2 searches. A total of 54.7% of complete BUSCO genes were duplicated, further supporting the Cat-4R duplication. RepeatModeler identified high levels of repeat content across the genome (53.98% of total assembly), including 21.49% DNA transposons (table 1).

**Table 1:** Genome Assembly Statistics

Assembly	Number of Contigs (initial assembly)	1,920
	Longest contig (initial assembly) (Mb)	28.88
	Contig N50 (initial assembly) (Mb)	4.19
	Final assembly length (Gb)	2.58
	Number of scaffolds (chromosomes)	50
	Number of contigs (final assembly)	904
	Contig N50 (final assembly) (Mb)	5.5
	Scaffold N50 (Mb)	49.2
	*N* (%)	0.003
	GC (%)	39.02
BUSCO v5	Complete (%)	98.3
(actinopterygii_odb10)	Complete single copy (%)	44.6
	Complete duplicated (%)	53.7
	Fragmented (%)	0.7
	Missing (%)	1.0
Repetitive elements	Total (%)	53.98
	SINEs (%)	0.25
	LINEs (%)	4.60
	LTR elements (%)	7.86
	DNA transposons (%)	21.49
	Unclassified (%)	19.77
Annotation	Predicted genes	57,229
	Mean [median] gene length (bp)	13,532.7 [8,018]
	Mean [median] exon length (bp)	195.9 [128]
	Mean [median] intron length (bp)	1,735.3 [541]
	Mean [median] exons per gene	7.9 [5]
	Mean [median] introns per gene	6.9 [4]

### Genome Architecture and Evolution

**Fig. 1.**
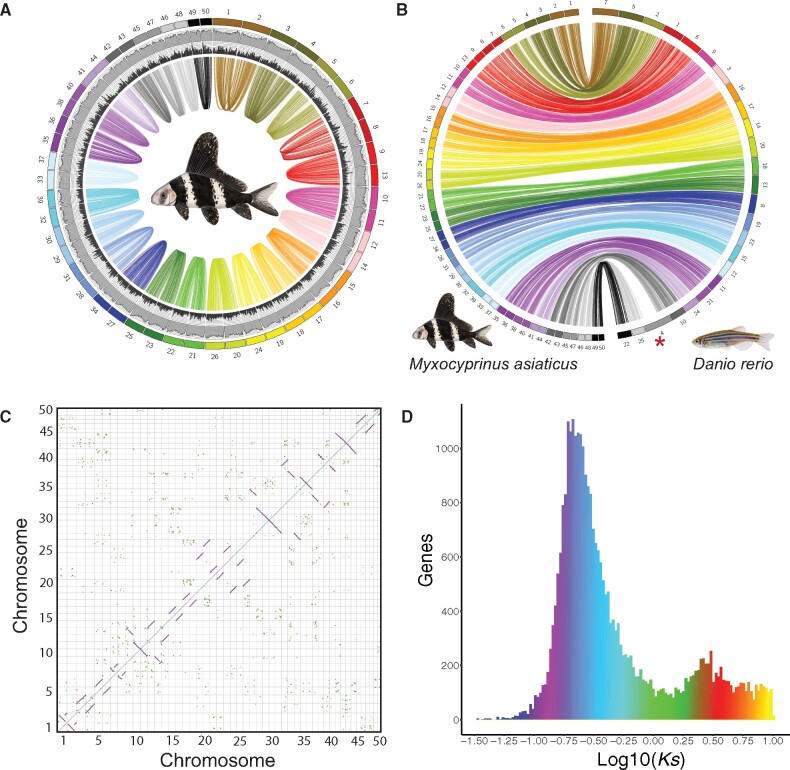
Circos plots illustrating *Myxocyprinus* self–self syntenic mapping (panel *A*) or *Myxocyprinus* versus zebrafish (panel *B*). Arcs reflect blocks with shared synteny between chromosomes and barplots in panel *A* reflect gene content (inner black ring) or repeats per MB (outer gray ring). Dotplot comparisons of *Myxocyprinus* self–self (panel *C*) and synonymous substitution histogram (*K_s_*; panel *D*). Panel *C* is a mirror image above and below the diagonal. The blue peak in panel *D* reflects the Cat-4R duplication, whereas the red peak represents the teleost 3R duplication; point colors in panel *C* correspond to panel *D* values. The red asterisk in panel *B* denotes the gap in synteny with the long arm of zebrafish chromosome 4, a zebrafish-specific sex-associated region.

MAKER3 annotations included a total of 57,229 predicted genes (table 1). We found 87% (49,687) of the predicted genes had at least one BLAST hit against a database of *D.rerio* peptides, with 68% (33,881) of hits >70% sequence similarity and >50 amino acid residues. Also, 69% (39,586) of the predicted genes contained a total of 82,674 putative protein domains. Genome annotation results, Circos plots and dotplots from self–self syntenic mapping revealed clear evidence of the Cat-4R genome duplication (fig. 1*A and C*). Homeologous chromosomes are readily apparent for all 25 pairs of chromosomes, with remarkably strong, conserved synteny across all pairs. Syntenic mapping between *Myxocyprinus* and zebrafish also showed remarkable stability of genome architecture (fig. 1*B*) between the two species despite around 100 Myr divergence between the two lineages (Bagley et al. 2018). Again, nearly all 25 chromosome pairs showed strong, shared synteny between the two species. The lone exception is the long arm of zebrafish chromosome 4, which lacks a homologous region in *Myxocyprinus* (fig. 1*B*, red asterisk). This arm contains a previously identified sex-associated region known to have arisen from a zebrafish lineage-specific expansion (Howe et al. 2013). Homeologous chromosomes tended to be remarkably similar in length, share similar numbers of annotated genes, and have similar numbers of repeats per megabase length (supplementary fig. S4, Supplementary Material online). These results suggest lack of obvious fractionation bias (i.e., loss of duplication genes and no clear subgenome dominance). Synonymous substitution plots (*K_s_*) of duplicate gene pairs also provide evidence for the Cat-4R WGD event. The large peak in the histogram around *K_s_ *=* *0.184 corresponds to the Cat-4R duplication (fig. 1*D*). Genes with *K_s_* values near this value tend to be found in the duplicated blocks in figure 1*C* (blue–purple colors). In addition to the Cat-4R peak, a smaller peak corresponding to the teleost-specific duplication (3R) is also seen in figure 1*D* (red colors). Given a mean estimate of 335 Myr since the 3R duplication and modal *K_s_* for the 3R peak of 2.441 we estimated a synonymous substitution rate of 0.007288 substitutions per site per million years. Using this rate, we estimate 25.2 Myr since the Cat-4R genome duplication.

In summary, the *Myxocyprinus* genome assembly provides a useful resource and displays remarkably strong, conserved synteny and genome architecture following a WGD event at least 25.2 Ma. Future work should focus on mechanisms maintaining this apparent genome stability and evaluate the extent of subgenome dominance, biased fractionation, and fate of duplicated genes after the Cat-4R duplication. Additionally, more work is needed to reconcile the Cat-4R time estimates with dates based on the fossil record and molecular phylogenetics (e.g., Bagley et al. 2018). This first catostomid genome assembly adds to a growing list of polyploid animal genomes to complement the wealth of research in plant and fungi systems.

## Materials and Methods

One juvenile *M.asiaticus* individual was obtained from Mystic Blue Aquarium Store (Buffalo, NY, USA; supplementary fig. S1, Supplementary Material online). The fish was sacrificed with an overdose of MS-222 and tissues were immediately dissected and placed in RNA-later and stored at −80 °C (supplementary table S1, Supplementary Material online). Total RNA was extracted using the RNA-Mini kit (Invitrogen Inc.) with Trizol and DNase treatment following the manufacturer’s recommendations. RNA from the 12 tissues was sent to Novagene, Inc. (Davis, CA, USA) for library preparation and mRNA sequencing on the NovaSeq6000 platform. Skeletal muscle tissue was mechanically homogenized in liquid nitrogen and high-molecular-weight (HMW) DNA was extracted using Qiagen Genomic-tip 500/G kit following the manufacturer’s protocol. HMW DNA was quantified on the Qubit 3.0 and used in ligation kit library preparation (LSK-109) and sequenced using 12 R9.4 flow cells on the Oxford Nanopore Technologies GridION platform. Base-calling was conducted using Guppy (v3.0.7; nanoporetech.com). Additionally, Illumina PE150 Novaseq6000 shotgun genomic reads were generated by Novogene (Davis, CA, USA) for assembly polishing.

### Genome Assembly and Scaffolding

The expected genome size was approximately 1.98 Gb (2.02 pg; Suzuki 1992) or 2.69 Gb (2.75 pg; Zhu et al. 2012) and with *n* = 50 chromosomes based on flow cytometry and karyotyping. Raw nanopore reads passing Guppy QC filters were filtered by discarding reads <1,000 bp. The remaining reads were concatenated and mapped pairwise using Minimap2 (v2.17; Li 2018) with the –x ava-ont flag. Miniasm (v0.3-r179; Li 2016) was used to produce an initial assembly based on the Minimap2 results. Consensus sequences from the Miniasm assembly were generated using two rounds of Racon (v1.4.11; Vaser et al. 2017) and polished with two rounds of Pilon (v1.23; Walker et al. 2014) using the Illumina reads. Purge_haplotigs (Roach et al. 2018) was used to identify and remove possible haplotigs and high coverage areas. Microbial and other contamination was searched for in the final assembly using Kraken2 (v2.1.0; Wood et al. 2019) using archaea, bacteria, plasmid, viral, human, fungi, plant, and protozoa databases. BUSCO (v5.1.2; Seppey et al. 2019) was used to assess completeness of the final assembly (without gene models) using actinopterygii_odb10 and to assess the relative fraction of duplicated genes. Dovetail Genomics Inc. (Scotts Valley, CA, USA) produced and sequenced Chicago and Hi-C libraries for genome scaffolding using skeletal muscle tissue as starting material.

### Transcriptome Assembly and Genome Annotation

Raw sequence data were run through TrimGalore (Martin, 2011) with default settings to remove all excess adaptors. The RNA-seq data was pooled across the 12 tissues and assembled de novo using the Trinity pipeline (v2.6.6; Grabherr et al. 2011) with the –trimmomatic flag. Gene models were generated using the MAKER3 pipeline (v.3.01.03 Holt and Yandell 2011; Campbell et al. 2014). First, repeats were identified and soft masked using RepeatModeler v2.0.1 (Flynn et al. 2020). Coding sequences from the Uniprot-SwissProt database (Poux et al. 2017) present in the custom repeat library were identified and discarded using BLAST (Altschul et al. 1990). The custom repeat library was combined with the RepBase v.23.09 vertebrate database (Bao et al. 2015) and a GFF file was generated using RepeatMasker v.4.1.1 (Smit et al. 2015). Additionally, we supplied MAKER3 with the transposable element library included with RepeatMasker for additional repeat masking. All repeats were soft-masked (softmask = 1) for annotation with MAKER3.

Three rounds of genome annotation were performed with MAKER3. First, we aligned evidence to the reference genome. For evidence, we supplied proteomes from two close relatives of the Chinese sucker: *C.carpio* (RefSeq GCF_000951615.1) and *D.rerio* (RefSeq GCF_000002035.6). We also used the de novo Trinity-assembled transcriptome as evidence. The first round of MAKER3 with evidence alignment was used to train the gene prediction software SNAP v2013-02-16 (Korf 2004) and Augustus v.19.12.2006 (Stanke et al. 2006). To avoid poor training performance, only gene models with a minimum length of 50 amino acids and a minimum annotation edit distance of 0.25 were included to train SNAP. Augustus training was performed using BUSCO v.3.1.0 (Simão et al. 2015), specifying zebrafish (*D.rerio*) as the initial gene model and using the BUSCO vertabrata_odb9 database.

One round of ab initio annotation with MAKER3 was performed using SNAP and Augustus gene models, SNAP and Augustus were retrained, and a final round of ab initio annotation was performed. Both rounds of ab initio gene prediction used all protein, RNA, and repeat alignments as evidence. Annotation statistics were generated using custom scripts and genestats (https://gist.github.com/darencard/fcb32168c243b92734e85c5f8b59a1c3; last accessed August 18, 2021). To identify putative functions of predicted proteins, a BLAST v.2.2.31+ (Camacho et al. 2009) search was performed using *D.rerio* peptides (RefSeq GCF_000002035.6) as the reference database. Results were filtered to include only matches with >70% sequence similarity and matches longer than 50 amino acid residues. In addition, InterProScan v.5.52-86.0 (Jones et al. 2014) was used to assign putative protein domain information to the predicted genes.

### Syntenic Mapping and *K_s_* Plots

A self–self syntenic map was produced using CoGe (Comparative Genomics, genomevolution.org; Lyons et al. 2008). CoGe’s SynMap2 was used to identify syntenic gene pairs with LASTZ searches and the DAGChainer algorithm (Haug-Baltzell et al. 2017), requiring a maximum distance between two matches (−D) = 20 and minimum of five aligned pairs (−A). Circos (Krzywinski et al. 2009) was used to generate plots based on synteny using the DAGchainer aligncoords file produced by CoGe SynMap2 (Haug-Baltzell et al. 2017) for self–self *Myxocyprinus* comparisons and *Myxocyprinus* versus zebrafish. In both instances, DAGChainer results were filtered to retain only one LASTz hit per query with the highest percent sequence identity. Dotplots were produced in a similar manner using CoGe SynMap2 and points were colored by mean synonymous substitution values (*K_s_*) for each syntenic gene. Synonymous substitutions were calculated using CodeML of the PAML package (Yang 2007), as implemented in CoGe.

## Supplementary Material

Supplementary data are available at *Genome Biology and Evolution* online.

## Supplementary Material

evab190_Supplementary_Data

## Data Availability

Data presented in this article are available from NCBI (Project PRJNA739167; BioSamples SAMN19774987-SAMN19774992).
