# Supplementary material for: Chromosome-Level Genome Assembly of Chinese Sucker (Myxocyprinus asiaticus) Reveals Strongly Conserved Synteny Following a Catostomid-Specific Whole-Genome Duplication
Source: Genome Biol Evol. 2021 Aug 12;13(9):evab190. doi: 10.1093/gbe/evab190 (PMC8412299; doi:10.1093/gbe/evab190)

**Supplemental Table 1.** Data used in genome assembly and polishing.

| Nanopore | sequences | 9,069,422 |
| --- | --- | --- |
|  | sum length (Gb) | 148.31 |
|  | minimum length (bp) | 38 (trimmed to 1,000) |
|  | average length (bp) | 16,353 |
|  | max length (bp) | 388,385 |
|  | N50 (bp) | 37,906 |
| Illumina | sequences | 1,348,688,218 |
|  | sum length (Gb) | 202.30 |
|  | read length (bp) | 150 |

**Supplemental Table 2.** *Myxocyprinus* RNA-seq data.

| **Tissue** | **Raw reads** | **Clean reads** | **Raw reads (Gb)** | **Clean reads (Gb)** |
| --- | --- | --- | --- | --- |
| Skeletal muscle | 22,427,639 | 22,157,558 | 6.7 | 6.6 |
| Gill | 22,452,758 | 21,922,026 | 6.7 | 6.6 |
| Heart | 22,245,587 | 21,797,182 | 6.7 | 6.5 |
| Swim bladder | 23,241,236 | 22,925,546 | 7.0 | 6.9 |
| Gall bladder | 21,006,637 | 20,634,626 | 6.3 | 6.2 |
| Eye | 21,696,283 | 21,482,317 | 6.5 | 6.4 |
| Brain | 24,353,870 | 23,983,358 | 7.3 | 7.2 |
| Skin | 24,964,693 | 24,448,971 | 7.5 | 7.3 |
| Scale | 21,673,795 | 21,335,782 | 6.5 | 6.4 |
| Spleen | 25,580,619 | 25,301,439 | 7.7 | 7.6 |
| Liver | 26,823,889 | 26,293,798 | 8.0 | 7.9 |
| Lips | 25,397,780 | 24,655,727 | 7.6 | 7.4 |

**Supplemental Table 3.** *Myxocyprinus* chromosome length, gene, and repeat content.

| **Chromosome** | **Length (bp)** | **Genes** | **Repeats** |
| --- | --- | --- | --- |
| 1 | 72239572 | 1698 | 71510 |
| 2 | 69913489 | 1578 | 69683 |
| 3 | 67839431 | 1585 | 69757 |
| 4 | 63939302 | 1567 | 66195 |
| 5 | 59718715 | 1662 | 61477 |
| 6 | 58241907 | 1558 | 59806 |
| 7 | 57435940 | 1317 | 56229 |
| 8 | 56796202 | 1279 | 58647 |
| 9 | 53982959 | 1267 | 56379 |
| 10 | 53891373 | 1132 | 56265 |
| 11 | 52806452 | 1088 | 55214 |
| 12 | 52518154 | 1454 | 54613 |
| 13 | 52423035 | 1198 | 54032 |
| 14 | 52350049 | 1414 | 54123 |
| 15 | 51769034 | 1247 | 53010 |
| 16 | 51521742 | 1306 | 53288 |
| 17 | 50994176 | 1130 | 52726 |
| 18 | 50364729 | 1159 | 49834 |
| 19 | 49695298 | 1039 | 50060 |
| 20 | 49533668 | 1159 | 51555 |
| 21 | 49270151 | 1011 | 51837 |
| 22 | 49154624 | 1050 | 49425 |
| 23 | 49034912 | 1047 | 51080 |
| 24 | 48473654 | 1017 | 48101 |
| 25 | 48452391 | 1109 | 50803 |
| 26 | 48241554 | 1147 | 50403 |
| 27 | 47945289 | 1157 | 49537 |
| 28 | 47583655 | 1185 | 48728 |
| 29 | 46545311 | 1215 | 47686 |
| 30 | 46486142 | 1315 | 48479 |
| 31 | 46382562 | 1121 | 48517 |
| 32 | 46271812 | 1067 | 46651 |
| 33 | 45607420 | 995 | 48466 |
| 34 | 45467162 | 1211 | 46320 |
| 35 | 44524880 | 948 | 46908 |
| 36 | 43596531 | 983 | 45819 |
| 37 | 43459174 | 964 | 45358 |
| 38 | 42813564 | 1079 | 45106 |
| 39 | 42260598 | 1000 | 44532 |
| 40 | 41621991 | 1113 | 41916 |
| 41 | 40590254 | 917 | 41870 |
| 42 | 39651316 | 1010 | 41265 |
| 43 | 38478457 | 973 | 40706 |
| 44 | 37057815 | 854 | 38621 |
| 45 | 34328074 | 815 | 35461 |
| 46 | 32896516 | 859 | 35254 |
| 47 | 31746090 | 792 | 31414 |
| 48 | 31011571 | 868 | 33579 |
| 49 | 28113276 | 781 | 30586 |
| 50 | 27735066 | 789 | 29713 |

**Supplemental Figure 1.** Chinese sucker, *Myxocyprinus asiaticus*, specimen used in genome sequencing.


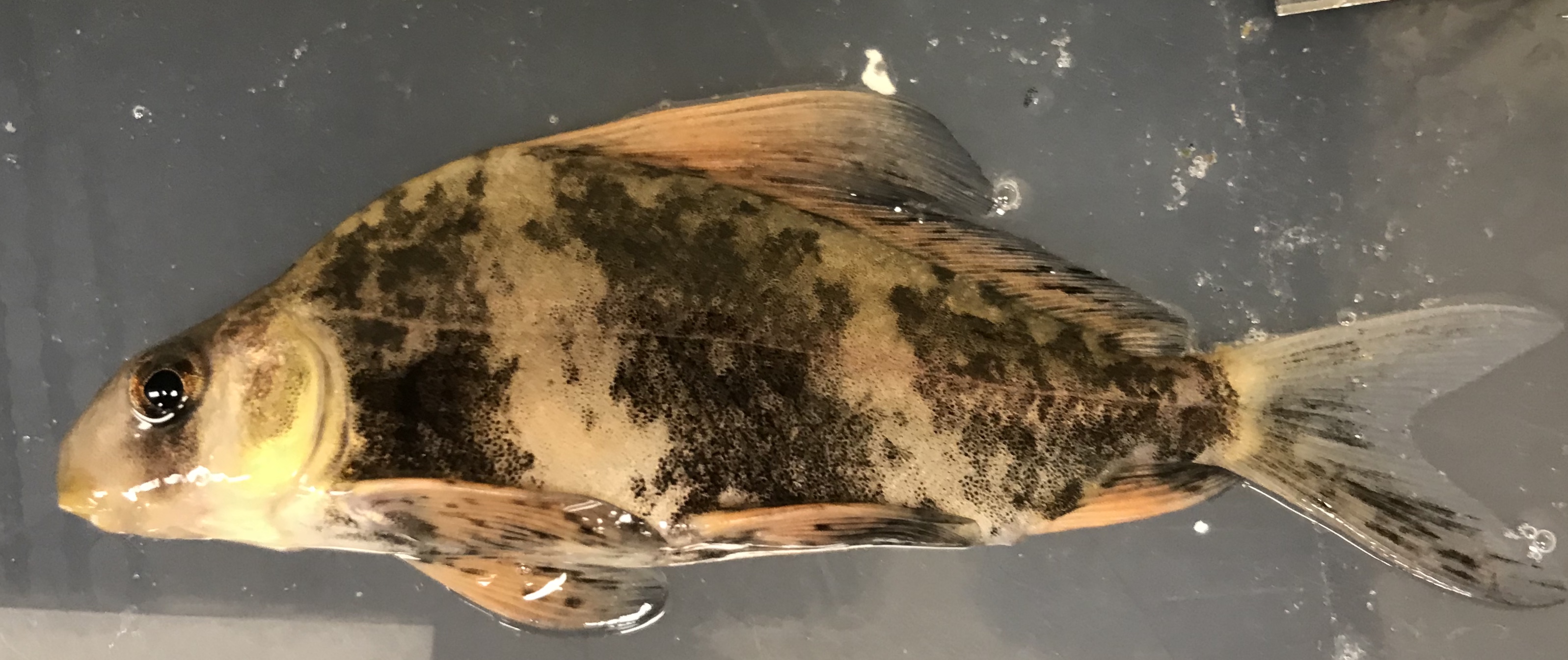


**Supplemental Figure 2.** Nanopore read length histogram. Vertical blue line represents read N50 (37,906 bp). The longest read was >388 kb.


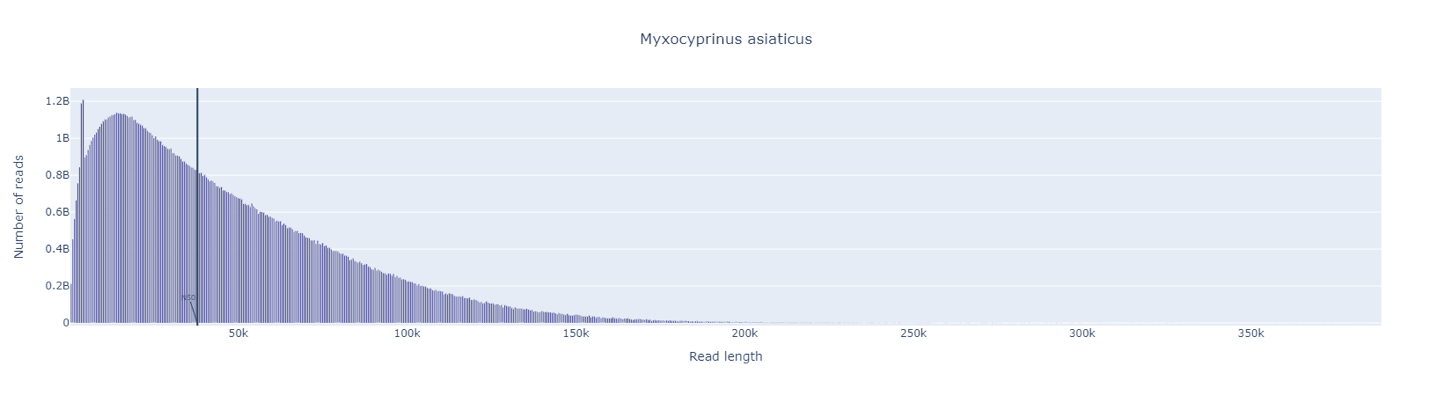


**Supplemental Figure 3.** BUSCO results on final assembly. A total of 54.7% of complete genes are duplicated, consistent with previous estimates from allozyme data from other catostomids (e.g., Ferris and Whitt, 1981).


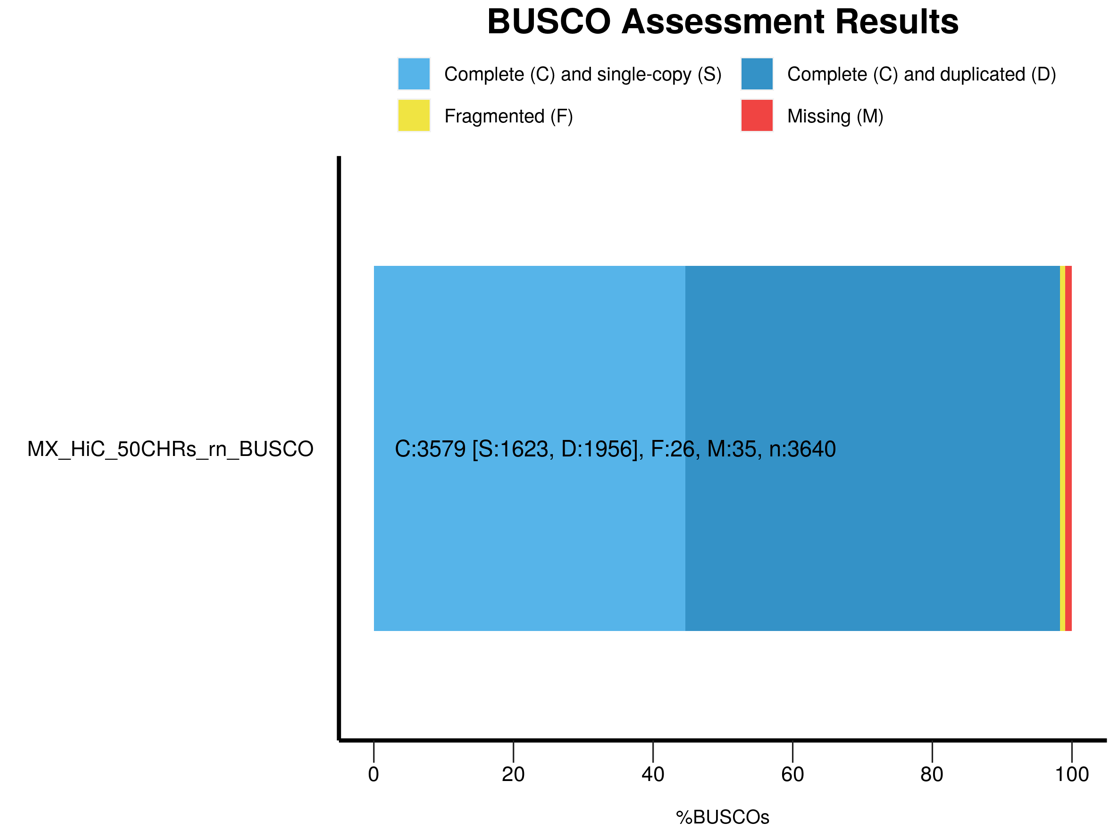


**Supplemental Figure 4.** Scatterplots of chromosome length (left), genes per chromosome (middle), and repeats per Mb (right) for homeologous chromosome pairs. Lines represent the 1:1 diagonal. Homeologous chromosomes tended to be remarkably similar in length (left panel). Similarly, a strong linear relationship between number of annotated genes was observed for homeolog pairs (middle panel), suggesting lack of obvious biased loss of duplication genes (“fractionation”). Repeat content was somewhat more variable among homeologous chromosome pairs (right panel), but no obvious bimodality was observed, indicating a lack of clear sub-genome dominance.


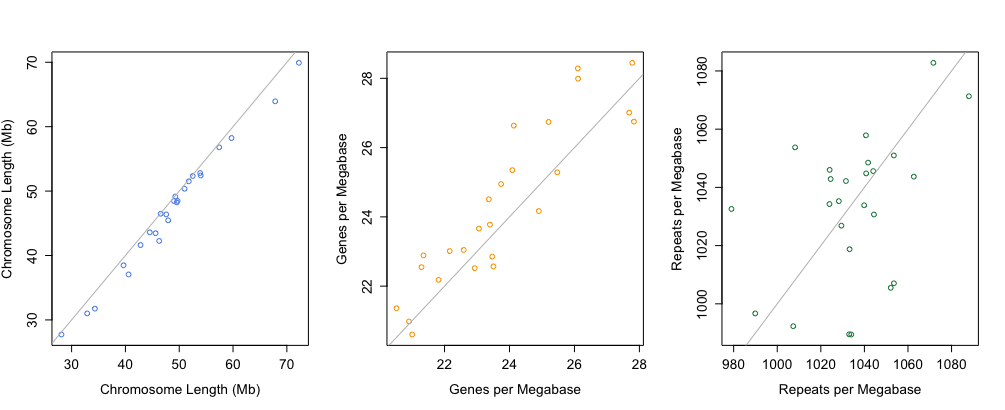

Supplement: evab190_Supplementary_Data [file evab190_supplementary_data.docx]
